# Comparative analysis of serum and saliva samples using Raman spectroscopy: a high-throughput investigation in patients with polycystic ovary syndrome and periodontitis

**DOI:** 10.1186/s12905-023-02663-y

**Published:** 2023-10-04

**Authors:** Dangli Hu, Jianmei Wang, Tianfan Cheng, Huijun Li, Feng Zhang, Dan Zhao, Xiaoyi Xu, Rong Yu, Ping Wen, Yunfei Cheng, Jian Xu, Lijian Jin, Jilong Yao

**Affiliations:** 1 Department of Obstetrics & Gynecology, Shenzhen Maternity and Child Healthcare Hospital, The First School of Clinical Medicine, Southern Medical University, Shenzhen, 518028 China; 2 Single-Cell Center, CAS Key Laboratory of Biofuels, Shandong Key Laboratory of Energy Genetics and Shandong Energy Institute, Qingdao Institute of Bioenergy and Bioprocess Technology, Chinese Academy of Sciences, Qingdao, 266101 China; 3 University of Chinese Academy of Sciences, Beijing, 101408 China; 4 Division of Periodontology & Implant Dentistry, Faculty of Dentistry, The University of Hong Kong, Hong Kong SAR, China; 5 Department of Stomatology, Shenzhen Maternity and Child Healthcare Hospital, Shenzhen, 518028 China; 6 Department of Implant Dentistry, Beijing Stomatological Hospital, Capital Medical University, Beijing, 100050 China; 7 Department of Science & Education, Shenzhen Maternity and Child Healthcare Hospital, Shenzhen, 518028 China

**Keywords:** Raman spectroscopy, Polycystic ovary syndrome, Periodontal diseases, Periodontitis, Metabolic indicators, Inflammatory mediators, Sex hormone

## Abstract

**Background:**

Polycystic ovary syndrome (PCOS) and periodontitis significantly affect women’s oral and systemic health worldwide, and yet increase the risk of cardiovascular-metabolic diseases like diabetes and coronary heart disease. Regarding the PCOS-periodontitis connection, whether sex hormones, metabolic and inflammatory mediators could account for the underlying linking mechanism needs to be further investigated. This case–control study evaluated the hormonal, metabolic and inflammatory profiles in PCOS and non-PCOS subjects with various periodontal conditions, via assessing serum and saliva samples by Raman spectroscopy.

**Methods:**

A total of 66 females with PCOS and 22 systemically healthy female volunteers were recruited in a single hospital. Full-mouth periodontal examination was undertaken for identifying the subjects with periodontal health, gingivitis or periodontitis. The datasets of sex hormones and metabolic indicators were retrieved from the hospital information system. Both serum and saliva samples were collected for detecting inflammatory mediators and Raman spectroscopic assessment. The subjects were categorized into four groups according to their conditions of PCOS and periodontitis for Raman spectroscopic analysis. Partial least squares discriminant analysis was performed to examine the inter-group differences in Raman spectra.

**Results:**

PCOS patients exhibited greater mean probing depth (*P* < 0.05) and higher serum levels of triglycerides (*P* < 0.05) and matrix metalloproteinase-8 (*P* < 0.05) than those in non-PCOS participants. Both probing depth and triglyceride level were positively correlated with PCOS (*P* < 0.05). There was a significant difference in mean Raman spectra of saliva samples among the four groups with different conditions of PCOS and periodontitis (*P* < 0.05), while no significant inter-group difference existed in serum samples.

**Conclusions:**

The present study shows that periodontal condition may affect the biomolecular profiles of Raman spectra in serum and saliva of PCOS patients. It underscores the importance of the collaborative teamwork of dentists and gynecologists for enhancing women’s oral health, general wellbeing and quality of life.

**Supplementary Information:**

The online version contains supplementary material available at 10.1186/s12905-023-02663-y.

## Background

Polycystic ovary syndrome (PCOS) as an endocrine metabolic disorder, badly affects the health of females at child-bearing age worldwide, with a prevalence of approximately 4 to 21% [1, 2]. The 2003 Rotterdam definition criteria are used widely to identify PCOS patients with at least two of the following features, including clinical and/or biochemical hyperandrogenemia, oligo- and/or anovulation as well as polycystic ovarian morphology on ultrasound [3]. Here, other causes of clinical and/or biochemical hyperandrogenemia (e.g., congenital adrenocortical hyperplasia, androgen-secreting tumours, and Cushing's syndrome) need to be excluded. Hyperandrogenemia is one of the crucial pathological features of PCOS, and it can lead to abnormal levels of other sex hormones, such as increased ratio of estrone to estradiol (E2) and a marked rise in the ratio of luteinizing hormone (LH) level to follicle stimulating hormone (FSH) [4]. Patients with PCOS may experience anovulatory infertility and exhibit an increased susceptibility to developing long-term cardiovascular outcomes and metabolic diseases like diabetes, hypertension, coronary heart disease and endometrial malignancy [5, 6].

Periodontal diseases, including gingivitis and periodontitis, are highly common inflammatory conditions in humans. Severe periodontitis affects 10–15% of the population globally, and it can result in substantial destruction of tooth-supporting structures and eventually severe tooth loss and edentulism [7]. In fact, the impact of periodontitis reaches far beyond the mouth, and it is closely associated with inflammatory systemic comorbidities such as diabetes, cardiovascular diseases and adverse pregnancy outcomes [8–10]. It has been shown that gingivitis is very common in patients with PCOS, and they exhibited an increased susceptibility to periodontitis [11]. Recently, the linkage of PCOS and periodontal diseases has been increasingly investigated and documented with elaboration of underlying clinical implications [12–16]. Moreover, Porwal et al. [17] have reported a high prevalence of gingivitis and periodontitis in women with PCOS than those without PCOS. A recent systematic review and meta-analysis reveals that PCOS patients exhibit a 28% increased risk of developing periodontitis, and yet those with periodontitis demonstrate a 46% increased risk of developing PCOS [15], suggesting that PCOS and periodontitis may be bidirectionally interconnected. Furthermore, studies [18–21] have found that the relationship between PCOS and periodontitis could be linked through the underlying mechanisms like a low chronic inflammatory status, oxidative stress, alterations and imbalances in the oral microflora as well as disturbances in endocrine metabolism. Indeed, it has been suggested that PCOS-related hormonal and metabolic disorders may increase the host susceptibility to periodontal diseases [22, 23]. PCOS could be particularly associated with the severity of periodontal inflammation via involving various inflammatory mediators like interleukin-6 (IL-6), IL-17 and matrix metal-loproteinase-8 (MMP-8) in serum and saliva samples [24–26]. The MMPs play an important role in regulating the whole process of follicle development [27]. It has been demonstrated that ovulatory dysfunction in PCOS patients is associated with the disturbances in serum MMP levels [28]. Thus, it may be speculated that the levels of MMP-8 could be associated with PCOS. However, the exact role of sex hormones in connection to the release of proinflammatory cytokines remains unknown.

Raman spectroscopy, being a noninvasive, rapid and label-free approach, can generate unique molecular fingerprints of various biological molecules [29]. This approach has been increasingly applied in biomedical science. Momenpour et al. [30] have shown that surface-enhanced Raman scattering combined with principal component analysis (PCA) could assess and profile the patients with PCOS. A recent review has concluded that Raman spectroscopy may be used to distinguish subgingival bacteria, analyze relevant changes in saliva, and identify bone transformation [31]. As such, Raman spectroscopy may have the potential value for assessing PCOS and periodontal diseases, while revealing the metabolic profiles underlying these two interconnected diseases.

The present study further evaluated the association of periodontal status with PCOS, via assessing the hormonal, metabolic and inflammatory profiles in PCOS and non-PCOS subjects with various periodontal conditions. Yet, this study explored for the first time the potential novel biomarkers for assessing PCOS and periodontal diseases, via analyzing Raman spectra of serum and saliva samples among these subjects. It was hypothesized that periodontal status may affect PCOS conditions and the related bio-molecular profiles in serum and saliva samples detectable by Raman spectroscopy. This work could contribute to enhancing the oral and women healthcare, through the close teamwork of dental and medical professionals.

## Methods

### Participants

This case–control study was undertaken in line with the Strengthening the Reporting of Observational Studies in Epidemiology guidelines [32]. A total of 107 Chinese patients diagnosed with PCOS (aged 20–34 years) following the 2003 Rotterdam definition criteria [3] and 22 systemically healthy female volunteers were recruited from both Departments of Gynecology and Stomatology at the Shenzhen Maternity & Child Healthcare Hospital (SZMCH) in Guangdong, China from October 2021 to August 2022. The diagnosis of PCOS was made on the basis of any two of the following criteria: i) clinical and/or biochemical hyperandrogenemia, such as signs of hirsutism, seborrheic alopecia, acne, and/or serum total testosterone (T) levels exceeding 7.5 μg/L; ii) menstruation < 8 times yearly or menstrual cycle > 35 days; and iii) transabdominal or transvaginal ultrasound showing a unilateral ovarian volume of ≥ 10 ml and/or ≥ 12 follicles of 2–9 mm diameter on the same surface of one ovary. Meanwhile, other conditions accounting for abnormal ovulation or hyperandrogenemia (e.g., congenital adrenocortical hyperplasia, androgen-secreting tumours, Cushing's syndrome, and thyroid abnormalities) were excluded. Systemically healthy female volunteers (aged 20–34 years) had menstrual cycles of 26–30 days, with normal results of medical examinations performed within one year other than metabolic disease (including insulin resistance, hypertension and hyperlipoidemia). Additionally, all regular sex hormone indicators were within the reference range 3 months prior to the enrolment. The following subjects were excluded, including i) taking antibiotics within 3 months and/or receiving periodontal treatments within 12 months; ii) systemically unhealthy prior to the diagnosis of PCOS or having systemic comorbidities other than metabolic complications following the diagnosis of PCOS; iii) receiving assisted reproductive technology; iv) continuously taking immunosuppressive agents, bisphosphonate osteoporosis drugs, steroids and immune-related drugs; and v) unwilling to participate in this study. In the sampling procedure, the non-PCOS subjects were selected to match the characteristics of PCOS patients, including age, body mass index (BMI) and waist-to-hip ratio (WHR), and then three PCOS patients per non-PCOS subjects were enrolled. Oral and informed written consent was received from all subjects prior to the study. This research work was approved by the Ethics Committee of SZMCH (No. SFYLS [2021]050), and the study was performed following the 2013 Declaration of Helsinki. The personal information of all the subjects and the obtained datasets were only used for scientific research and not disclosed to the public.

### Demographic and clinical datasets

At the time of enrolment, the following information and datasets were obtained from all participants through interviews and questionnaires, including demographic characteristics (age and education attainment), lifestyles (smoking, drinking and sleep problems), gestation records, history of PCOS (diagnosis, duration and treatments), psychological status (depression, anxiety and stress), oral hygiene practice (regular dental visit, symptom of gingival bleeding during brushing, awareness of periodontal diseases), other oral/periodontal conditions (periodontal abscess and endodontic periodontal lesions, mucogingival deformities, occlusal trauma and dental prosthesis) and general health status. Psychological health status was evaluated by the Depression Anxiety and Stress Scale-21 [33]. The modified Ferriman-Gallwey (mFGS) scoring system [34] was used to evaluate the severity of hirsutism, one of the hyperandrogenic symptoms in patients with PCOS. Physical examination was performed to record BMI, WHR as well as systolic and diastolic blood pressure (SBP and DBP). The results of various serum biochemical parameters, including triglycerides (TG), total cholesterol (TC), high-and low-density lipoprotein cholesterol (HDL and LDL), fasting plasma glucose (FPG) and levels of LH, FSH, T, prolactin (PRL), progesterone (P) and E2 were retrieved from the dataset of SZMCH.

### Periodontal conditions

All subjects received full-mouth periodontal examination at 6 sites of each tooth by a single calibrated examiner (HJL), using a UNC-15 probe (Hu Friedy, USA). All examinations were employed in line with the concurrent time points of physical and biochemical check-ups. The intra-examiner reliability was evaluated by repeated measurements at the site level in four subjects, with the intraclass correlation coefficient of 0.887 (95% CI: 0.860–0.909) for the full agreement on probing depth (PD) and 0.873 (95% CI: 0.829–0.905) on clinical attachment loss (CAL). All examinations were undertaken in line with the concurrent time points of medical check-ups. The documented periodontal parameters consisted of full-mouth plaque score (FMPS, %), bleeding on probing (BOP, %), PD, tooth mobility, number of remaining teeth and number of tooth losses due to periodontitis (excluding the third molars). CAL was calculated with reference to PD and gingival recession. On the same day, the subjects also underwent panoramic X-rays for assessing alveolar bone levels. To avoid the possible effects on embryo in case of pregnancy, all the subjects were suggested to use contraception for the menstrual cycle after radiographic examination. Subsequently, the subjects were generally identified as non-periodontitis (periodontal health and gingivitis) and periodontitis groups, following the current classification of periodontal diseases [35–38].

### Collection of saliva and serum samples

**Fig. 1:**
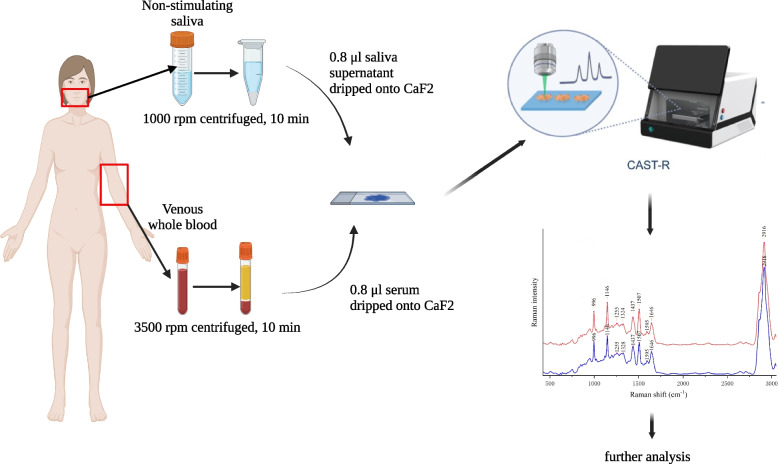
Flow chart of Raman spectroscopy imaging for serum and saliva samples (Created with BioRender.com)

All samples were collected in the morning following a 12-h fast within the early follicular phase (Days 2 to 5) of the spontaneous or progesterone-induced menstrual cycle (Fig. 1). Prior to oral examination, the participants were instructed not to drink (except water) or take chewing gum. Approximately 5 ml of unstimulated whole saliva samples were collected, and then centrifuged at 1,000 rpm for 10 min at 4 °C. The supernatant was transferred to a 500 μL storage tube and stored at -80 °C for further analysis. Venous blood (5 mL) was obtained from the antecubital vein following the standard venipuncture. The serum samples were obtained by centrifugation (3,500 rpm for 10 min at 4 °C), transferred to a 500 μL storage tube and stored at -80 °C until the biochemical analysis was performed.

### Measurement of inflammatory parameters

Enzyme-linked immunosorbent assay (ELISA) kits (Finetest, Guangzhou Chenxue Biotech Co., Ltd., China) were used to quantitate the levels of IL-6, IL-17A and MMP-8 in both saliva and serum samples.

### Acquisition of Raman spectra

After thawing at room temperature and appropriately mixing, 0.8 μL of the saliva samples were taken and deposited as a drop on the calcium fluoride substrate at room temperature. In addition, 2 μL of serum samples were mixed with 14 μL of ultrapure water in an Eppendorf tube. Then, 0.8 μL of the mixture was taken and deposited as a droplet on a CaF2 substrate at room temperature. Subsequently, Raman spectra were acquired from the Clinical Antimicrobial Susceptibility Test Ramanometry system (CAST-R, Qingdao Single-cell Biotechnology, Qingdao, Shandong, China) equipped with a 532 nm Nd: YAG laser, a 100 × air objective (Olympus, Japan) with a numerical aperture of 0.9 nm and a spectral resolution of ∼2 cm^−1^ for the observation of body fluids. In the pre-experiments, the Raman spectroscopy parameters were first provided based on the previous experience of the specialist technicians in the detection of serum and saliva samples, and then adjusted according to the signal-to-noise ratio and stability of the obtained Raman spectroscopy signals. The parameters of the equipment currently displayed for the detection of saliva and serum samples were finally determined. The laser power was 100 mW. The spectra ranged from 420 to 3050 cm^−1^, and the accumulative times of a single point were one time using a 5/1 s acquisition time (saliva/serum samples). Ten scattered points of each sample from the ring region were selected to minimize the bias in testing based on the preliminary experimental results.

The flow chart is presented in Fig. 1 and Figure S1.

### Statistical analysis

The sample size was estimated using software (G*Power 3.1.9.7). Based on the results of the study by Dursun et al. [11] on PCOS patients, a considerable effect value (d > 0.80) was expected. Therefore, the difference in PD values of 0.50 ± 0.3 mm between the PCOS and non-PCOS groups was used as a reference. Setting a significance level of 5% and detection power of 85%, the sample size was considered to be at least 62 (1:1). To account for the unequal distribution between the groups (1:3), a sample size of at least 82 participants was determined.

The non-Raman spectroscopy data mainly include demographic information, anthropometric parameters, hormonal and metabolic indicators, periodontal parameters and the levels of inflammatory mediators (IL-6, IL-17A and MMP-8). The results were presented appropriately (mean ± SD or median with IQR for continuous variables, and frequency for categorical variables). Intergroup differences in continuous variables were evaluated by *t*-tests or Mann–Whitney *U* tests, while categorical variables were examined with the χ^2^ tests. Binary logistic regression was employed to analyze the odds ratio (OR) for assessing the possible influence of clinical indicators on periodontitis. Intergroup analysis of variability and logistic regression models was undertaken to examine the relationship among PCOS and periodontal conditions and hormonal/metabolic indicators. The statistical analyses were undertaken with a software tool (SPSS version 26, IBM Corporation, NY, USA). A two-sided *P* < 0.05 was deemed to be statistically significant. Considering the potential risks of false positive results, the statistical significances was adjusted as 0.05/6 = 0.0083 for IL-6, IL-17A and MMP8 as well as 0.05/7 = 0.0071 for T, P, E2, PRL, LH, LH/FSH and FSH, respectively.

The raw Raman spectrum was presented as a signal map with a high individual variability requiring certain processing prior to subsequent analysis. Labspec 5 software (HORIBA Jobin Yvon Ltd., U.K.) was then used to preprocess the Raman spectroscopy datasets following wavelet baseline correction and vector normalization steps. After importing the raw Raman data into the software, the ‘baseline correction’ and ‘normalization’ buttons were clicked on sequentially, and the preprocessed Raman spectra were obtained. Ten spectra were recorded, preprocessed and averaged for each sample to generate a representative, stable and reliable spectrum for further analysis. Average spectra were statistically assessed and graphed using Origin2021 (Version 9.80). After obtaining stable and reliable salivary Raman spectra, literature searching for characteristic peaks with high repetition rates in the same group of Raman spectra for attribution was helpful for analyzing the components of different groups. The differences among multiple groups for selected peaks of Raman spectra were appropriately assessed using analyses of variance (ANOVA) or the Kruskal‒Wallis test. Partial least squares discriminant analysis (PLS-DA) was conducted to evaluate the differences between the spectra of the same sample. The spectra were then classified and distinguished accordingly. PCA was used to analyze the differences in Raman spectra between samples and grouping factors. Permutational multivariate analysis of variance was performed to analyze the extent to which different grouping factors could account for the difference in samples, and statistics analysis was performed using permutation tests (two-sided *P* < 0.05 was considered statistically significant). The receiver operating characteristic (ROC) curve was employed to demonstrate the predictive ability of the model for different disease states. Spearman correlation analysis was used to explore the correlations among the clinical indicators, inflammatory mediators, sex hormones and metabolic indicators, periodontal parameters and Raman spectroscopic results. Internal scripts were used for further analysis and visualization under the R environment (Version 4.2.2). The ‘mixOmics’ package in R (Version 4.2.2) was employed for above analysis. The relevant script code is available at http://mixomics.org/ (Table S1).

## Results

### Clinical dataset of subjects with different periodontal conditions

**Fig. 2:**
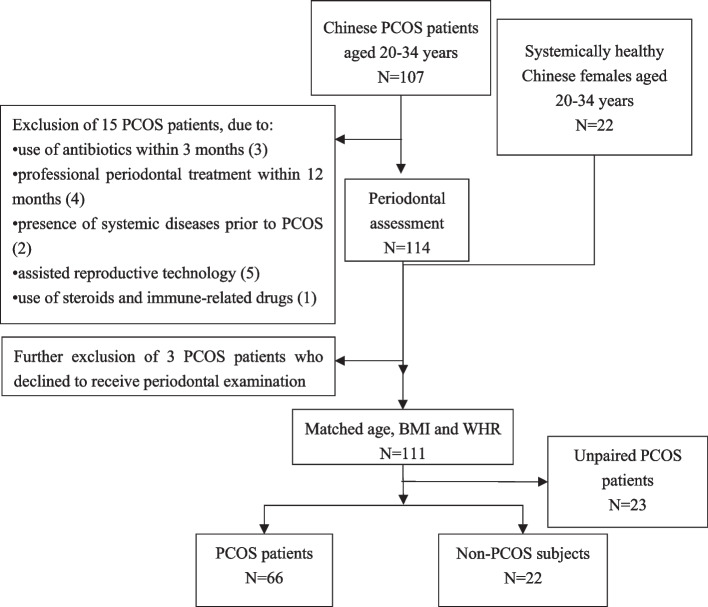
Flow chart of the 88 participants included in the study

After assessing and matching the 129 initially recruited participants, a total of 66 PCOS patients (26.0 ± 3.3 years) and 22 healthy subjects (25.7 ± 3.5 years) were finally included in the data analysis (Fig. 2). Just over half (56.8%) of the subjects showed a monthly household income of over 9,000 CNY, 75.0% received college education or above, and 30.7% of them had regular dental visits (Table S2). It is worth noting that 31.8% exhibited mild to moderate periodontitis (15.9% at Stages I and II, respectively). The rest were non-periodontitis subjects including gingivitis (36.4%) and periodontal health (31.8%). More than four-fifths (80.7%) presented over 50% of sites with plaque, and 62.5% with 10–50% of BOP sites.

The datasets of demographic, anthropometric, periodontal and inflammatory indicators from the subjects with or without PCOS are presented in Table 1. Overall, there were similar demographic profiles and lifestyles in these two groups. As anticipated, notable inter-group difference existed in the levels of LH (*P* < 0.001), T (*P* < 0.001), E2 (*P* < 0.05) and LH/FSH (*P* < 0.001). While there were no significant differences in the levels of FSH, PRL and P between the two groups. Notably, mean PD was significantly higher in PCOS patients than that in non-PCOS participants (*P* < 0.05). Whereas, there was no notable difference in the presence of periodontitis between PCOS and non-PCOS subjects. Moreover, MMP-8 level in serum samples was significantly higher in PCOS patients than that in the controls (*P* < 0.01). In addition, as shown in Table S3, salivary MMP-8 level was significantly higher in periodontitis group than that in non-periodontitis group (*P* < 0.001).

**Table 1:** Comparison in demographic, anthropometric, periodontal and inflammatory indicators between the patients with and without PCOS

Parameters	PCOS (*n* = 66)	Non-PCOS(*n* = 22)	*P* value
Age (years)	26.0 ± 3.3	25.7 ± 3.5	0.782
Education			0.394
Lower than university level	18(27.3)	4(18.2)	
University level or higher	48(72.7)	18(81.8)	
Income (CNY)			0.456
< 9,000	27(40.9)	11(50)	
≥ 9,000	39(59.1)	11(50)	
Smoking			0.409
Never	64(97.0)	22(100.0)	
Previously	2(3.0)	0(0.0)	
Drinking			0.698
Never	58(87.9)	20(90.9)	
Seldom	8(12.1)	2(9.1)	
Regular dental visit			0.505
No	47(71.2)	14(63.6)	
Yes	19(28.8)	8(36.4)	
Dietary habits			0.795
Fat-reduced or normal meals	43(65.2)	15(68.2)	
High-fat meal	23(34.8)	7(31.8	
BOB			0.596
No	39(59.1)	13(59.1)	
Yes	27(40.9)	9(40.9)	
BMI (kg/m2)	22.66 ± 3.91	21.63 ± 2.96	0.261
WHR	0.78 ± 0.05	0.77 ± 0.04	0.347
SBP (mmHg)	116.6 ± 11.6	115.7 ± 12.3	0.758
DBP (mmHg)	72.2 ± 8.7	69.4 ± 9.7	0.201
HR (bpm)	86.1 ± 12.6	80.5 ± 13.0	0.076
mFGS	2.0 ± 1.5	1.0 ± 1.3	0.005
PD (mm)	1.67(1.55, 2.09)	1.52(1.46, 1.75)	0.022
Percentage of sites with PD ≥ 4 mm (%)	0(0,3.0)	0(0,1.3)	0.296
Percentage of sites with PD ≥ 6 mm (%)	0(0,0)	0(0,0)	0.564
FMPS (%)	73.0(57.0, 88.3)	63.5(51.5, 86.0)	0.325
BOP (%)	14.5(7.7, 33.0)	10.7(7.7, 21.6)	0.208
Alveolar bone resorption (%)^a^	15.0(14.0,20.0)	15.0(13.5,21.5)	0.855
Periodontal status			0.290
no periodontitis	43(65.2)	17(77.3)	
periodontitis	23(34.8)	5(22.7)	
FSH (IU/L)	6.99 ± 2.20	7.43 ± 2.51	0.438
LH (IU/L)	14.94 ± 7.37	4.87 ± 1.91	< 0.001
LH/FSH	2.05(1.63,2.67)	0.58(0.46,0.84)	< 0.001
T (μg/L)	0.77(0.67,0.86)	0.52(0.44,0.62)	< 0.001
PRL (μg/L)	15.30(9.62,19.95)	13.34(9.07,20.94)	0.802
P (μg/L)	0.77(0.43,1.24)	0.79(0.29,1.00)	0.388
E2 (pg/mL)	43.91(32.03,73.75)	33.30(25.90,45.25)	0.014
TG (mmol/L)	0.88(0.64,1.43)	0.68(0.59,0.85)	0.033
TC (mmol/L)	4.87 ± 0.89	4.62 ± 0.78	0.249
LDL (mmol/L)	2.67(2.41,3.22)	2.67(2.47,3.11)	0.603
HDL (mmol/L)	1.47 ± 0.41	1.41 ± 0.33	0.496
FPG (mmol/L)	5.00 ± 0.51	5.03 ± 0.36	0.816
se IL-6 (pg/mL)	2.16(1.03,3.49)	2.96(1.98,4.03)	0.068
se IL-17A (pg/mL)	30.26(11.32,814.86)	23.43(16.55,539.75)	0.413
se MMP-8 (ng/mL)	5.13(3.30,9.70)	1.97(1.38,4.79)	0.001
sa IL-6 (pg/mL)	2.47(1.24,4.18)	2.44(0.85,4.92)	0.908
sa IL-17A (pg/mL)	10.05(6.34,13.87)	9.83(8.00,16.94)	0.446
sa MMP-8 (ng/mL)	153.13(54.02,326.50)	120.00(34.01,240.41)	0.350

Data are presented as mean ± SD or median (IQR) or frequency (%)

^a^The percentage of alveolar bone resorption is only presented in periodontitis subjects, including 23 PCOS patients and 5 non-PCOS subjects

Binary logistics regression models were used to analyze the potential links of sex hormones, metabolism, inflammatory indicators and periodontal parameters with PCOS conditions (Table 2). Notably, the levels of TG, PD and mFGS were positively correlated with PCOS (OR: 4.108, *P* < 0.05; 7.027, *P* < 0.05; and 1.806, *P* < 0.01; respectively).

**Table 2:** Binary logistic regression on the association of hormonal, metabolic, inflammatory and periodontal parameters with PCOS status

Variates	β	OR (95%CI)*	*P*-value
TG	1.413	4.108 (1.056, 15.989)	**0.042**
PD	1.950	7.027 (1.180, 41.848)	**0.032**
mFGS	0.591	1.806(1.157,2.818)	**0.009**

^*^OR (95% CI) was examined by binary logistic regression analysis, including mFGS, HR, PD, TG, E2, se IL-6, se MMP-8 via a backward stepwise approach. Boldfaces are used to show the significance when *P* < 0.05

### Analysis of serum and saliva samples by Raman spectroscopy

**Fig. 3:**
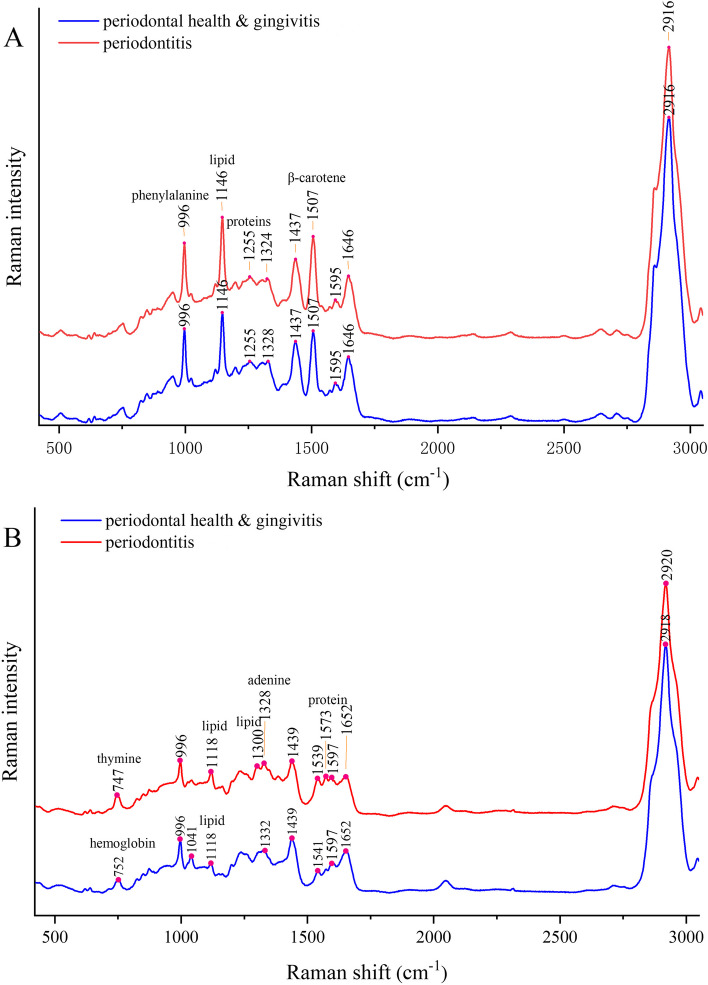
Average Raman spectra of samples under different periodontal conditions. **A** Ramen spectra of serum samples: Peaks from left to right: 996- phenylalanine [39], 1146- carbon–carbon bonding modes from lipids [43], 1255- amide III [43], 1324- guanine, adenine [41], 1437- collagen, 1507- carotenoids [44], 1595- cytosine, 1646- amide I band, and 2916- lipid methyl or methylene stretch [44]; **B** Raman spectra of saliva samples: Peaks from left to right: 747- hemoglobin, 752- hemoglobin, 996- phenylalanine, 1041- glycated protein [44], 1118- carbon–carbon bonding modes stretch from lipids [43], 1300- fatty acids, 1328- guanine, adenine [41], 1332- phenylalanine, 1439- collagen, 1539- carotenoids, 1541- carotenoids, 1573- protein, 1597- cytosine [44], 1652- amide I band [40] and 2920- lipid [44]

Overall, the Raman spectra ranged from 423 cm^−1^ to 3050 cm^−1^, and 88 averaged spectra in each type of sample were obtained after preprocessing. Figure 3A shows the average Raman spectra of serum samples from subjects with non-periodontitis (periodontal health and gingivitis) and periodontitis. The major peaks in the serum samples were observed at approximately 996 cm^−1^, 1146 cm^−1^, 1255 cm^−1^, 1324–1328 cm^−1^, 1437 cm^−1^, 1507 cm^−1^, 1595 cm^−1^, 1646 cm^−1^ and 2916 cm^−1^ Raman shifts in both statuses (Table S4). The peak at 996 cm^−1^ was attributed to aromatic ring breathing, especially phenylalanine [39]. The peak at 1146 cm^−1^ was mainly assigned to the carbon‒carbon bonding mode of lipids, while the peaks at 1255 cm^−1^, 1324 cm^−1^, 1328 cm^−1^ and 1646 cm^−1^ were attributed to protein and amino acid vibrations [40–43]. Moreover, the peak at 1507 cm^−1^ was related to carotenoids [44] with various clinical implications such as cancer treatment, cardiovascular disease prevention, cataract, antioxidant and anti-ageing effects [45]. The peak at 2916 cm^−1^ had the highest frequency value and corresponded to lipid structures and methyl stretching vibrations [44].

The average Raman spectra of saliva samples from subjects with different periodontal statuses are presented in Fig. 3B. The major peaks in saliva samples were observed at approximately 996 cm^−1^, 1118 cm^−1^(lipid), 1439 cm^−1^(collagen), 1597 cm^−1^ and 1652 cm^−1^ (protein and amide I band) Raman shifts in both statuses [40–44]. There were some peaks in different statuses, such as 1300 cm^−1^(fatty acids), 1573 cm^−1^(protein) and 2920 cm^−1^(protein), which were only present under the periodontitis condition.

**Fig. 4:**
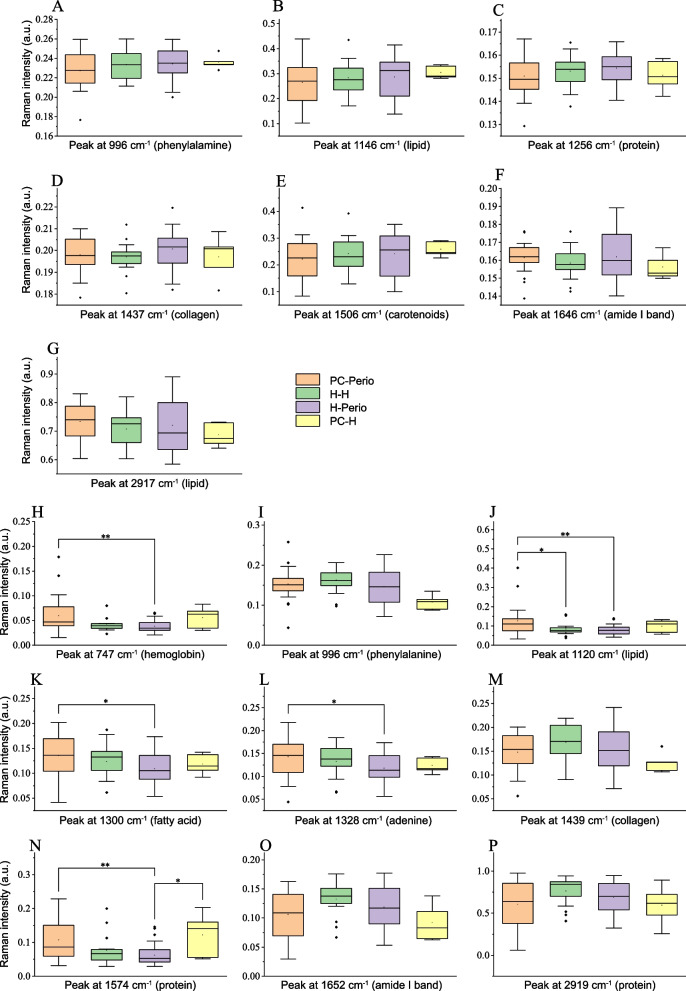
Pairwise comparison of major peaks in serum and saliva samples of the four groups. **A**-**G** Major peaks of Ramen spectra in serum samples; The selected peaks in serum samples revealed no notable difference among the 4 groups; **H**-**P** Major peaks of Ramen spectra in saliva samples; The mean Raman intensities of the major peaks at 747 cm^−1^ (hemoglobin), 1120 cm^−1^ (lipid), 1300 cm^−1^ (Fatty acids), 1328 cm^−1^ (adenine) and 1574 cm.^−1^ (protein) were higher in the PC-Perio group than those in the H-Perio group (*P* < 0.05). Inter-group difference of continuous variables was determined by Bonferroni-corrected ANOVA or Kruskal–Wallis test as appropriate. *P < 0.05; **P < 0.001

To further investigate the effect of PCOS and periodontitis on the different Raman spectra, all the subjects were categorized as non-PCOS and non-periodontitis group (H–H, *n* = 17), PCOS and non-periodontitis group (PC-H, *n* = 43), non-PCOS and periodontitis group (H-Perio, *n* = 5), and PCOS and periodontitis group (PC-Perio, *n* = 23). Figure 4 reveals the peaks of serum and saliva samples presented in Fig. 3. The mean Raman intensities of the major peaks in the serum samples were not significantly different among the four groups. However, there were significant intergroup differences with salivary Raman peaks at 747 cm^−1^, 1120 cm^−1^, 1300 cm^−1^, 1328 cm^−1^ and 1574 cm^−1^ (*P* < 0.05). The mean Raman intensities of the significant peaks at 747 cm^−1^ (hemoglobin), 1120 cm^−1^ (lipid), 1300 cm^−1^ (fatty acids), 1328 cm^−1^ (adenine) and 1574 cm^−1^ (protein) were all higher in the PC-Perio group than those in the H-Perio group (*P* < 0.05). The mean Raman intensity at 1120 cm^−1^ (lipid) was higher as well in the PC-Perio group than that in the H–H group (*P* < 0.05). The peaks at 1574 cm^−1^ (protein) had greater mean Raman intensities in the PC-H group than that in the H-Perio group (*P* < 0.05).

**Fig. 5:**
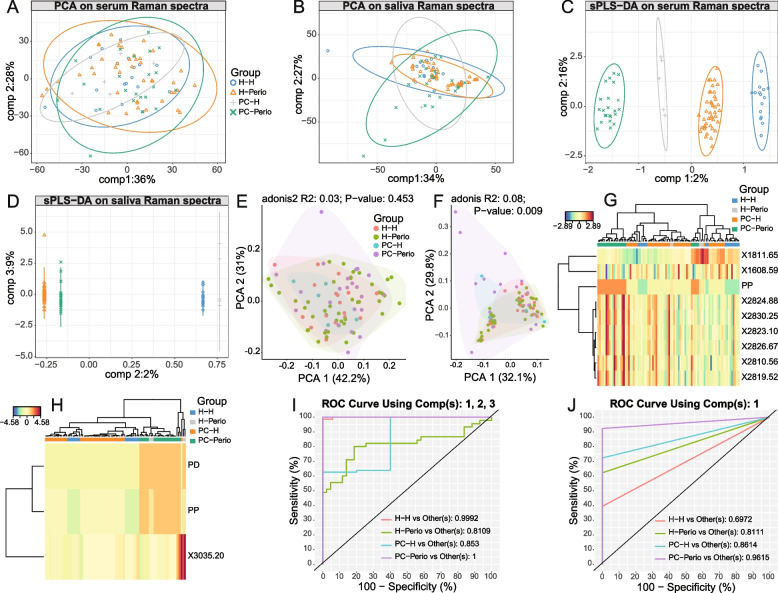
PLS-DA analyses and ROC curves in both serum and saliva samples under different periodontal conditions. **A** **C** score plots of serum Raman spectra after PCA and PLS-DA analysis for PC1 and PC2 dimensions in H–H, PC-H, H-Perio and PC-Perio groups; **G** heat map of serum Raman spectra after PLS-DA analysis for cluster analysis of the selected PC; **E** adonis test for intergroup difference after PLS-DA for serum Raman spectra; **I** ROC curves to analyze the ability of salivary Raman spectra to discriminate between PCOS and periodontitis; **B**, **D** score plots of saliva Raman spectra after PCA and PLS-DA analysis for PC1 and PC2 dimensions in H–H, PC-H, H-Perio and PC-Perio groups; **H** heat map of saliva Raman spectra after PLS-DA analysis for cluster analysis of the selected PC; **F** adonis test for intergroup difference after PLS-DA for saliva Raman spectra; **J** ROC curves to analyze the ability of serum Raman spectra to discriminate between PCOS and periodontitis

To verify whether the differences detected may contribute to the establishment of a classification model for discriminating the signals collected from serum and saliva samples, PCA and PLS-DA were then performed on the collected spectra. After PCA analysis, the four groups were clustered to a certain extent, and each sample was distributed fairly evenly within its own 95%CI range, with no obvious discrete points (Fig. 5A and B). A PLS-DA classification model analysis of the Raman spectra of the serum samples revealed no aggregation among the four groups and a more concentrated distribution of samples within each group (Fig. 5C), similar to the Raman spectra of the saliva samples (Fig. 5D). There was no marked inter-group difference in the Raman spectra of the serum samples (*R*^2^ = 0.03, *P* = 0.453) (Fig. 5E). However, there was a significant difference among the four groups in the saliva samples (*R*^2^ = 0.08, *P* < 0.01) (Fig. 5F). Figure 5G and H showed that there could also have influences on the components between samples of the same type. However, the same groups of Raman spectra from the same sample type tended to cluster into one group and showed more similarity. The aggregation effect of grouping was more evident for saliva samples than that for serum samples. ROC analysis further indicated that the area under the curve (AUC) of saliva samples was more than 0.069 (*P* < 0.001).

### Correlation of clinical parameters with the results of Raman spectra

Most of the major peaks related to periodontal status and PCOS belonged to lipids and proteins (Table S4). The differences in periodontal parameters, sex hormone levels and cytokine levels between the PCOS and periodontitis groups are presented in Table 1 and Table S3. It was concerned that these two diseases could be interlinked with changes in certain chemical compounds. Therefore, Spearman correlation tests were performed for related hormones and cytokines as well as the average Raman intensities corresponding to proteins and lipids (Tables S5 and S6).

The most significantly correlated components existed in the H-Perio group for both serum and saliva samples, followed with the PC-Perio group and the least in the H–H group. These associations, most positively, occurred mainly between hormones and cytokines. In the PC-H group, the level of IL-6 in serum was positively correlated with BOP, PD and Raman average spectral intensity of proteins (value = 0.9, *P* < 0.05). The levels of T in PC-H group were positively correlated with the levels of MMP-8 (value = 0.9, *P* < 0.05). The levels of MMP-8 in serum were also positively correlated with BOP (value = 0.462, *P* < 0.05) and PD (value = 0.538, *P* < 0.01) in PC-Perio group. The mean salivary Raman spectral intensity attributed to proteins in the H-Perio group was positively correlated with LH/FSH (value = 0.314, *P* < 0.05) and negatively correlated with the level of IL-17A in saliva (value = -0.373, *P* < 0.05). The mean salivary Raman spectral intensities of the attributed lipids were positively correlated with LH/FSH (value = 0.357, *P* < 0.05) in H-Perio group, and the mean salivary Raman intensity of the attributed proteins (value = 0.450, *P* < 0.01) in PC-Perio groups, and negatively correlated with IL-17A (value = -0.374, *P* < 0.05) and BOP (value = -0.363, *P* < 0.05) in H-Perio group. However, in the PC-H group, the mean salivary Raman spectral intensities of the attributed lipids were negatively correlated with the levels of MMP-8 (value = -0.900, *P* < 0.05).

## Discussion

Regarding the relationships between PCOS and periodontitis, there are several interesting studies [46–50]. P may account for increased periodontal inflammation by affecting the vascular distribution in periodontal tissues, whereas E2 accounts for the inflammatory condition of the gingival cells [51, 52]. As such, there may exist a synergistic promoting effect on the occurrence and progression of gingivitis and periodontitis. Another review [48] shows that androgens affect periodontal diseases, through influencing bone metabolism and expression of inflammatory mediators in periodontal tissues. It is known that LH and FSH produced by the pituitary act on the corresponding receptors in the ovary, affecting the production and secretion of E2, P and T [46, 47]. It can be considered that LH may affect the development of periodontal inflammation via disturbing the secretion of E2, P and T. However, in the present study, there was no significant difference in sex hormone indicators between Periodontitis group and non-Periodontitis group that is inconsistent with those of previous studies. It is possibly due to the limited sample size of this study, which cannot fully reflect the characteristics of the overall cohort of PCOS patients. The subjects in the present study were relatively young and did not suffer from severe periodontitis. Further study is required to clarify this finding.

Poor oral hygiene often leads to the symptoms of BOB, the notable sign of periodontal inflammation [53, 54]. Periodontitis begins with gingivitis, a highly common inflammation of the gingiva caused by dysbiotic plaque biofilms and the disrupted immuno-inflammatory response [7]. In the present study, the proportion of BOB in periodontitis group was significantly higher than that in non-periodontitis group, in line with the current evidence.

Various inflammatory mediators, such as IL-6, IL-17A and MMP-8, are essentially involved in the pathogenesis of both PCOS and periodontitis, and especially, the expression and release of MMP-8 increase significantly during the aggravation of periodontal inflammation and the resultant destruction [25, 55]. MMP-8 is notably indicative of periodontal inflammation, and it is crucially involved in the progression of periodontitis and promotes the development of periodontal diseases at the active site of periodontal inflammation [55]. Therefore, MMP-8 is currently considered as one of the important biomarkers of periodontal inflammation and periodontitis. In this study, MMP-8 levels in serum samples were greatly higher in PCOS patients than those in non-PCOS participants, and yet salivary MMP-8 levels were markedly higher in periodontitis patients than those of non- periodontitis subjects. Taken together, these findings suggest that MMP-8 in serum may be positively correlated with PCOS, and those in saliva is indicative of periodontitis.

It is known that IL-6, IL-17A and MMP-8 are assigned to characteristic peaks of proteins in the Raman spectra [56, 57]. Interestingly, the average Raman intensities of the peaks at 747 cm^−1^ (hemoglobin) and 1574 cm^−1^ (protein) are higher in the PC-Perio group than those of the H-Perio group, suggesting that PCOS may affect the hemoglobin in periodontitis patients. Here, hemoglobin may be mainly derived from gingival bleeding, and therefore PCOS could increase the inflammation occurring in periodontitis patients [58, 59]. In addition, hemoglobin is one type of proteins containing iron [60]. Thus, PCOS may have influence on the metabolism of proteins and iron in periodontitis patients as well. Indeed, Spearman correlation analysis further demonstrates that the protein levels in the Raman spectra are positively correlated with the levels of IL-6. As such, the peaks at 747 cm^−1^, 1328 cm^−1^ and 1573 cm^−1^ in the saliva Raman spectra may be potential biomarkers for evaluating periodontal conditions. Furthermore, the Raman intensity levels of lipids and proteins are positively correlated. As mentioned above, PCOS is positively correlated with periodontitis, in both lipid and protein Raman spectra profiling. The PLS-DA results highlight that it is possible to identify periodontitis and non-periodontitis in PCOS patients by analyzing the Raman spectra of saliva samples. There were various significant correlations among periodontal parameters, sex hormones, cytokines as well as Raman spectra of proteins and lipids in serum and saliva samples. For instance, protein Raman average spectral intensity in serum is only positively correlated with levels of P in H–H group, IL-6 in PC-H group, and IL-17A in PC-Perio group. These findings suggest that periodontitis could affect the levels of biomolecules in serum and saliva of PCOS patients. Whereas, it is challenging to distinguish periodontitis and non-periodontitis with serum samples alone, as serum compounds are often affected by various systemic conditions and related confounders. Additionally, PCOS is an endocrine and metabolic disorder that significantly impacts serum components. Taken together, the present work suggests that the detection of salivary components by Raman spectroscopy may be a novel approach to assessing periodontal conditions for early detection of diseases and effective healthcare.

Some limitations of the present study need to be elaborated. This case–control study only shows the correlation between periodontitis and PCOS in Chinese young women with relatively high educational and economic levels from a maternal and child healthcare hospital, which may reduce the generalization of the current findings. Longitudinal studies with appropriate control of confounding factors should be undertaken, to clarify the exact inter-relationship between periodontitis and PCOS, and elaborate the potential clinical implications. Large sample-size investigations with representative cohorts and different environmental backgrounds should be conducted to provide more robust and generalizable evidence. Furthermore, it is highly expected that further study would explore the underlying molecular mechanisms of the PCOS-periodontitis connection, via multiomic approaches such as microbiomics, metabolomics and proteomics.

Notably, this study is the first attempt to use Raman spectroscopy to analyze serum and saliva samples in PCOS patients with different periodontal conditions. It is essential to emphasize that PCOS is linked with periodontitis at the average intensity level of Raman spectra, which is correlated with sex hormone and cytokine levels as well. It demonstrates that periodontitis may have influence on PCOS patients in term of proteins and iron metabolic profiles. Thus, Raman spectroscopy can serve as a noninvasive and rapid approach to assessing periodontal condition and metabolic profiles in PCOS patients on a long-term basis, thereby contributing to early detection and treatment of periodontitis as well as effectively managing PCOS patients for oral health and general wellbeing.

It could be pointed out that the present study mainly deals with the relationship between abnormalities in gynecological endocrinology and periodontal condition in young women of childbearing age. Therefore, the current results on the relationship of PCOS with periodontal diseases may differ from those from a larger population. It is hoped to raise the awareness of oral health for this specific group of young females to reduce the burden of periodontal diseases as early as possible. It is therefore strongly suggested that obstetricians and gynecologists need to pay more attention to periodontal status and oral care for patients with PCOS, and thereby arrange necessary referral for early and effective professional oral care. Meanwhile, oral health professionals should pay attention to the metabolic state of young women at childbearing age with periodontal diseases, and refer them timely to a gynecological clinic as appropriate.

## Conclusions

Within the limitations of the present work, this case–control study indicates that periodontal status could affect the biomolecular profiles in serum and saliva of young PCOS patients detectable by Raman spectroscopy in a fast and sensitive manner. This study suggests that periodontal health status could favorably contribute to modulating the metabolic, inflammatory and endocrine conditions in PCOS patients. Further multi-centered studies with large sample cohorts are highly warranted to provide more generalizable evidence on the PCOS-periodontitis interlink. It is of great importance to proactively integrate oral/periodontal healthcare in the long-term management of PCOS patients. Hopefully, the collaborative teamwork of dentists and gynecologists could greatly enhance women’s oral health, general health and quality of life in the near future.

### Supplementary Information

**Additional file 1: Document S1.** Questionnaire for subjects.

**Additional file 2: Document S2.** Inflammatory mediator testing protocol.

**Additional file 3: Figure S1.** Flow chart of the whole procedure of subject recruitment, examination and sample measurement.

**Additional file 4: Table S1.** The relevant script codes for R analysis.

**Additional file 5: Table S2.** Demographic data, anthropometric and periodontal indicators in all 88 subjects.

**Additional file 6: Table S3.** Comparison of demographic data, anthropometric, hormonal and inflammatory indicators between the periodontitis and non-periodontitis groups.

**Additional file 7: Table S4.** Attribution of the major peaks obtained from Raman analysis of serum and saliva samples (± 8 cm^−1^).

**Additional file 8: Table S5.** Spearman correlation analysis of hormonal, inflammatory and periodontal indicators, and proteins and lipids Raman spectra in serum samples.

**Additional file 9: Table S6.** Spearman correlation analysis of hormonal, inflammatory and periodontal indicators, and proteins and lipids Raman spectra  in saliva samples.

## Data Availability

The data presented in this study are openly available in Figshare with the identifier https://doi.org/10.6084/m9.figshare.23056766.v1.

## Abbreviations

ANOVA: Analyses of variance
AUC: The area under the receiver operating characteristic curve
BMI: Body mass index
BOB: Gingival bleeding during brushing
BOP: Bleeding on probing
CI: Confident interval
DBP: Diastolic blood pressure
E2: Estradiol
FMPS: Full mouth plaque score
FPG: Fasting plasma glucose
FSH: Serum follicle stimulating hormone
HDL: High-density lipoprotein cholesterol
H–H: Non-PCOS and non-periodontitis group
H-Perio: Non-PCOS and periodontitis group
HR: Heart rate
IL-6: Interleukin-6
IL-17A: Interleukin-17A
IQR: Interquartile range
LDL: Low-density lipoprotein cholesterol
LH: Luteinizing hormone
mFGS: Modified Ferriman-Gallwey score
MMP-8: Matrix metalloproteinase-8
OR: Odds ratio
P: Progesterone
PC: Principal component
PCA: Principal Component Analysis
PD: Probing depth
PC-H: PCOS and non-periodontitis group
PLS-DA: Partial Least Squares Discriminant Analysis
PC-Perio: PCOS and periodontitis group
PRL: Serum prolactin
ROC: Receiver operating characteristic
sa: Saliva
SBP: Systolic blood pressure
SD: Standard difference
se: Serum
SZMCH: The Shenzhen Maternity & Child Healthcare Hospital
T: Serum total testosterone
TC: Total cholesterol
TG: Triglyceride
WHR: Waist-to-hip ratio
