# Supplementary material for: Comparative analysis of serum and saliva samples using Raman spectroscopy: a high-throughput investigation in patients with polycystic ovary syndrome and periodontitis
Source: BMC Womens Health. 2023 Oct 4;23:522. doi: 10.1186/s12905-023-02663-y (PMC10552415; doi:10.1186/s12905-023-02663-y)
Supplement: Supplementary file 1 — Additional file 1: Document S1. Questionnaire for subjects. [file 12905_2023_2663_MOESM1_ESM.docx]

**Study of periodontal health and polycystic ovary syndrome in women**

**preparing for pregnancy**

In this questionnaire, we would like to provide you with some basic information about yourself, your illness and your lifestyle and eating habits. Please read the description and the questions carefully and answer them according to your actual situation. We promise that your information will not be disclosed or used in any way other than for this survey!

1. Your name is *

2. Your age is (years)*

3. Your own mobile phone number is *

4. Are you a Chinese national? *

○No (Please skip to the end of the questionnaire and submit your answer)

○Yes

5. Your level of education is *

○Junior high school and below

○High School and College

○University and above

6. Your current monthly household income: *

○Under ¥9,000

○¥9000~¥19999.99

○¥20,000 and above

7. Are you currently pregnant? *

○No

○Yes

8. Do you have any plans to become pregnant in the next year? *

○No

○Yes

9. Have you ever been pregnant or given birth in the past? (including biochemical

pregnancy, miscarriage)*

○No (Please skip to question 16)

○Yes

10. How many pregnancies have you had in the past? (including biochemical pregnancy,

miscarriage)

11. How many miscarriages have you had in the past? (including biochemical

pregnancies, not abortions)

12. How many deliveries have you had in the past? (after 28 weeks of pregnancy)

13. Whether you have had recurrent premature births or miscarriages, adverse pregnancy outcomes (e.g. miscarriage, fetal chromosomal abnormalities, embryonic abortion) in

previous pregnancies.

○Never happened

○Yes, it has happened 1-2 times

○Yes, ≥3 times

14. Your specific adverse pregnancy and childbirth outcomes are (for each pregnancy).

- Biochemical abortion
- Spontaneous abortion
- Induced abortion
- embryonic abortion
- Fetal chromosomal abnormalities
- Fetal malformation
- Abortion
- Premature birth (28-36+6 weeks gestation)
- Low birth weight babies (<2500g)
- Other

15. Any pregnancy complications or complications (e.g. hypertensive disorders of

pregnancy, gestational diabetes, etc.).

○No

○Gestational diabetes or diabetes combined with pregnancy

o Hypertensive disorders in pregnancy

○Intrahepatic cholestasis syndrome during pregnancy

○Pregnancy combined with haematological disorders (e.g. thrombocytopenia)

- Pregnancy combined with immune system disorders (e.g. systemic lupus erythematosus)
- Yes, other specific disease names

16. Your age at menarche is (years): *

17. Do you have regular periods without medication? *.

○No

○Yes

18. Average number of days of your menstrual period: *

○1-3 days

○4-10 days

○10 days or more

19. Your average menstrual cycle: *

o Less than 21 days, specific days

○21-35 days, specific days

○More than 35 days, specific days

20. Date of your last menstrual period: *

21. Do you have diabetes, thalassaemia, cardiovascular disease, cancer or tumours (except fibroids), infectious diseases (e.g. hepatitis B, syphilis, AIDS, etc.) or diseases of

the immune system (e.g. lupus erythematosus, rheumatoid arthritis, etc.)?

o None of the above

○Yes, please specify your current medication/hospital: (Please

skip to the end of the questionnaire to submit your answer)

o For other diseases, please specify current medication / hospital:

22. Do you have a history of genetic disorders? *

○No

○Yes, please specify: (Please skip to the end of the questionnaire

to submit your answer)

23. Do you have any congenital diseases (e.g. congenital heart disease, etc.)? *

○No

○Yes, please specify: (Please skip to the end of the questionnaire

to submit your answer)

24. Do you have any developmental abnormalities (e.g. ductus arteriosus, rickets, etc.) *.

○No

○Yes, please specify: (Please skip to the end of the questionnaire

to submit your answer)

25. Do you suffer from any of the following inflammatory diseases? *

○No

○Chronic pneumonia or bronchitis (Please skip to the end of the questionnaire and submit your answer)

- Urinary tract infections, e.g. chronic nephritis, nephrotic syndrome, etc. (Please skip to the end of the questionnaire to submit your answer)
- Chronic gastritis (Please skip to the end of the questionnaire to submit your answer)

o Recurrent vaginitis (Please skip to the end of the questionnaire to submit your answer)

○Other, please specify: _ (Please skip to the end of the

questionnaire to submit your answer)

26. Do you require ongoing immunosuppressive drugs, bisphosphonates for osteoporosis or hormonal and immune-related drugs such as steroids? (e.g. prednisone,

methylprednisolone etc)*.

○No

○Yes, please specify the medication taken and why: (Please skip

to the end of the questionnaire and submit your answer)

27. Have you had a history of taking antibiotics in the last 3 months?

○No (Please skip to question 30)

○There are

28. Type of antibiotics you take:

29. Your reasons for taking antibiotics.

30. when you were diagnosed with polycystic ovary syndrome*

31. How long have you been treating polycystic ovary syndrome with medication *

32. Do you have any medical conditions that predate the diagnosis of polycystic ovary

syndrome (e.g. diabetes, hypertension, etc.) *

○No

○Yes, please specify:

33. Do you take medication to treat your polycystic ovaries? *

○No

○Yes, taking only sex hormone based medication (estrogen and progestin)

○Yes, combined with a combination of Chinese and Western medicines (including proprietary Chinese medicines)

○Yes, take only herbal treatment

34. Have you developed complications of polycystic ovary syndrome (obesity, insulin

resistance, dyslipidaemia, hypertension, abnormal glucose tolerance, occurring later than the diagnosis of polycystic ovary syndrome) *

○No

○Yes, please specify complications:

○>2 times/year

35. Do you have hyperandrogenic manifestations (acne, hair loss, hirsutism, seborrheic

dermatitis, elevated serum testosterone, etc.)*

○No

○Yes, please specify the performance:

36. Do you have ultrasound findings of polycystic ovarian changes?

○No

○Yes

37. Do you know anything about periodontal disease and oral hygiene care? *

○No

○Yes

38. Have you had any periodontal treatment (e.g. scaling, etc.) in the last 12 months? *

○No

o Yes, please specify:

39. Do you have regular oral check-ups and periodontal treatment? *

○No

○1 time / 2 years

○1 time/year

○2 times/year

40. Does your family member suffer from periodontal disease? *

○No

o Yes, please specify

○Unknown

41. How often do you brush your teeth per day: *

○1 time

○2 times

○>2 times

42. Does the toothpaste you use contain fluoride: *

○No

○Yes

43. Do your gums bleed when you brush your teeth: *

○No

○Yes

44. Do you use dental cleaning products (e.g. floss, interdental brushes): *

○No

○Flossing

○Tooth gap brush

○Flossing and interdental brushes

45. How often do you use the dental cleaning products (times/day): *

46. Do you have any other oral cleaning habits (e.g. use of mouthwash, flosser, etc.): *

○No

○Yes

47. specific other oral cleansing modalities: , frequency

(times/day):

48. Do you actively smoke (at least 1 cigarette per day for more than 6 months): *

o Never smoked (Please skip to question 52)

○Previous smoker who has quit (at least 3 months in a row)

○Smoking (Please skip to the end of the questionnaire and submit your answer)

49. How often have you actively smoked in the past:

Note:Occasionally <3 times/week, frequently 3-4 times/week, always 5-6 times/week.

○Occasionally

○Often

○Always

○daily

50. Your previous monthly cigarette consumption (cigarettes/month).

51. How long have you smoked in the past:

smoking since you quit:_

and how long have you been

52. Are you a passive smoker (exposed to others smoking in your living or workplace for

more than 15 minutes at least one day a week)?

○No

○Yes

53. How often do you smoke passively.

<3 times/week occasionally, 3-4 times/week often, 5-6 times/week always.

○Occasionally

○Sometimes

○Often

○daily

54. How long have you been a passive smoker?

55. Do you have a long-term drinking habit?

○No (Please skip to question 58)

○Past drinker but abstaining from alcohol

○Occasionally

○Yes (Please skip to the end of the questionnaire and submit your answer)

56. What is the duration of your drinking?

57. The types of alcohol you drink are

Note: Occasionally <3 times/week, often 3-4 times/week, always 5-6 times/week.

- White wine, frequency and monthly consumption (ml/month):
- Wine, frequency and monthly consumption (ml/month):
- Foreign alcohol, frequency and monthly consumption (ml/month):
- Beer, frequency and monthly consumption (ml/month):
- Yellow wine, frequency and monthly consumption (ml/month):
- Alcoholic beverages, frequency and monthly consumption (ml/month):

58. The average number of hours you sleep per day*

○<6 hours

○6-8 hours

○>8 hours

59. How is the quality of your sleep ? *

○Not good, frequent insomnia and wakefulness

○Ordinary, occasional insomnia, wakefulness

○Very good, no insomnia or wakefulness

60. Do you stay up late (sleep later than 22:00)

Note: Occasionally <2 times/week, often 2-3 times/week, always 4-5 times/week

○No

○Occasionally

○Often

○Always

61. In the last 3 months, what was the main structure of your diet? *

○Low salt and low fat diet

○Low-carb, low-fat diet

○High protein, low carb, low fat diet

○High-carb, high-fat, heavy-salt diet

○Other, please specify _

62. How often do you take physical exercise: *

○Never

○Rarely (less than 1 time/week)

○Sometimes (1-3 times/week)

○Frequently (4-6 times/week)

○daily

63. the time you spend exercising each time: *

○<30 minutes

○30 minutes - 1 hour

o≥1 hour

64. Your height (cm): *

65. Your weight (kg): *

66. Waist circumference (cm).

67. Hip circumference (cm).

1. I find it hard to keep myself quiet

Based on the past week, choose the degree option that applies to your situation in each entry.

Rating level: 0 - does not fit; 1 - sometimes fits; 2 - often fits; 3 - always fits

○0

○1

○2

○3

2. I feel thirsty

Level of evaluation: 0 - does not fit; 1 - sometimes fits; 2 - often fits; 3 - always fits

○0

○1

○2

○3

3. I don't seem to feel any pleasure or relief at all

Level of evaluation: 0 - does not fit; 1 - sometimes fits; 2 - often fits; 3 - always fits

○0

○1

○2

○3

4. I feel breathless (e.g. shortness of breath or breathlessness)

Level of evaluation: 0 - does not fit; 1 - sometimes fits; 2 - often fits; 3 - always fits

○0

○1

○2

○3

5. I find it difficult to take the initiative to start working

Level of evaluation: 0 - does not meet; 1 - sometimes meets; 2 - often meets; 3 - always meets

○0

○1

○2

○3

6. I tend to react allergically to things

Level of evaluation: 0 - does not conform; 1 - sometimes conforms; 2 - often conforms; 3 - always conforms

○0

○1

○2

○3

7. I feel trembling (e.g., shaking hands)

Level of evaluation: 0 - does not fit; 1 - sometimes fits; 2 - often fits; 3 - always fits

○0

○1

○2

○3

8. I feel that I consume a lot of energy

Level of evaluation: 0 - does not fit; 1 - sometimes fits; 2 - often fits; 3 - always fits

○0

○1

○2

○3

9. I worry about occasions when I might panic or make a fool of myself

Level of evaluation: 0 - does not conform; 1 - sometimes conforms; 2 - often conforms; 3 - always conforms

○0

○1

○2

○3

10. I feel I have nothing to look forward to in the near future

Level of evaluation: 0 - does not match; 1 - sometimes matches; 2 - often matches; 3 - always matches

○0

○1

○2

○3

11. I feel apprehensive

Level of evaluation: 0 - does not fit; 1 - sometimes fits; 2 - often fits; 3 - always fits

○0

○1

○2

○3

12. I find it difficult to relax myself

Level of evaluation: 0 - does not fit; 1 - sometimes fits; 2 - often fits; 3 - always fits

○0

○1

○2

○3

13. I feel depressed and frustrated

Level of evaluation: 0 - does not fit; 1 - sometimes fits; 2 - often fits; 3 - always fits

○0

○1

○2

○3

14. I cannot tolerate anything that prevents me from continuing to work

Level of evaluation: 0 - does not conform; 1 - sometimes conforms; 2 - often conforms; 3 - always conforms

○0

○1

○2

○3

15. I feel on the verge of collapse

Level of evaluation: 0 - does not match; 1 - sometimes matches; 2 - often matches; 3 - always matches

○0

○1

○2

○3

16. I can't develop enthusiasm for anything

Level of evaluation: 0 - does not conform; 1 - sometimes conforms; 2 - often conforms; 3 - always conforms

○0

○1

○2

○3

17. I don't feel very worthy of being a human being

Level of evaluation: 0 - does not fit; 1 - sometimes fits; 2 - often fits; 3 - always fits

○0

○1

○2

○3

18. I find myself easily offended

Level of evaluation: 0 - does not conform; 1 - sometimes conforms; 2 - often conforms; 3 - always conforms

○0

○1

○2

○3

19. I feel an irregular heart rhythm even when I am not physically active in any significant way

Level of evaluation: 0 - does not fit; 1 - sometimes fits; 2 - often fits; 3 - always fits

○0

○1

○2

○3

20. I feel scared for no reason

Level of evaluation: 0 - does not fit; 1 - sometimes fits; 2 - often fits; 3 - always fits

○0

○1

○2

○3

21. I feel that life is meaningless

Level of evaluation: 0 - does not fit; 1 - sometimes fits; 2 - often fits; 3 - always fits

○0

○1

○2

○3
