# Supplementary material for: Comparative analysis of serum and saliva samples using Raman spectroscopy: a high-throughput investigation in patients with polycystic ovary syndrome and periodontitis
Source: BMC Womens Health. 2023 Oct 4;23:522. doi: 10.1186/s12905-023-02663-y (PMC10552415; doi:10.1186/s12905-023-02663-y)
Supplement: Supplementary file 2 — Additional file 2: Document S2. Inflammatory mediator testing protocol. [file 12905_2023_2663_MOESM2_ESM.docx]

**Measurement of inflammation parameters**

The levels of MMP-8, IL-17 and IL-6 in saliva and serum samples were quantified by enzyme-linked immunosorbent assay (ELISA) using the commercial ELISA kits (Finetest, Guangzhou Chenxue Biotech Co., LTD, China). Quantify the concentrations of the three inflammatory mediators in serum and saliva samples frozen at -80°C in accordance with the Human ELISA Kit instructions. The procedure was as follows:

1) Sample preparation: Take out the serum and saliva samples which are stored in the freezer at -80°C, leave them at room temperature for half an hour and then centrifuge them at 1000 rpm for 20 minutes to extract the supernatant. At the same time, all reagents in the kit are placed at room temperature for 20 minutes.

2) Prepare the working solution of the standards in multiples of 1:1, 1:2, 1:50, 1:100, 1:200 and 1:500 dilutions.

3) Add 400 μl of washing solution to each well of the enzyme plate, soak for 2 minutes, aspirate the liquid from the wells, pat dry on blotting paper and repeat this step 1 time.

4) Add 50 μl per well of the standard working solution and the test sample to each well of the ELISA plate in sequence. 50 μl of biotinylated antibody working solution is added immediately afterwards, mix gently, covered and incubated at 37°C for 45 minutes.

5) Remove the liquid and add 400μl of washing solution to each well, soak for 2 minutes, aspirate as much liquid as possible from each well, pat dry on absorbent paper and repeat this step 2 times.

6) Add 100μl of enzyme conjugate working solution to each well, cover with membrane and incubate at 37°C for 30 minutes.

7) Remove the liquid and add 400μl of washing solution to each well, aspirate as much liquid as possible from each well, pat dry on blotting paper and repeat this step 4 times.

8) Add 90μl of the substrate solution to each well of the enzyme plate, cover with a membrane and incubate at 37°C for 10-20 minutes.

9) Terminate the reaction by adding 50μl of termination solution to each well.

10) Immediately read the optical density value of each well at a wavelength of 450nm using an enzyme marker and calculate the final concentration.
