# Supplementary material for: Comparative analysis of serum and saliva samples using Raman spectroscopy: a high-throughput investigation in patients with polycystic ovary syndrome and periodontitis
Source: BMC Womens Health. 2023 Oct 4;23:522. doi: 10.1186/s12905-023-02663-y (PMC10552415; doi:10.1186/s12905-023-02663-y)
Supplement: Supplementary file 3 — Additional file 3: Figure S1. Flow chart of the whole procedure of subject recruitment, examination and sample measurement. [file 12905_2023_2663_MOESM3_ESM.docx]

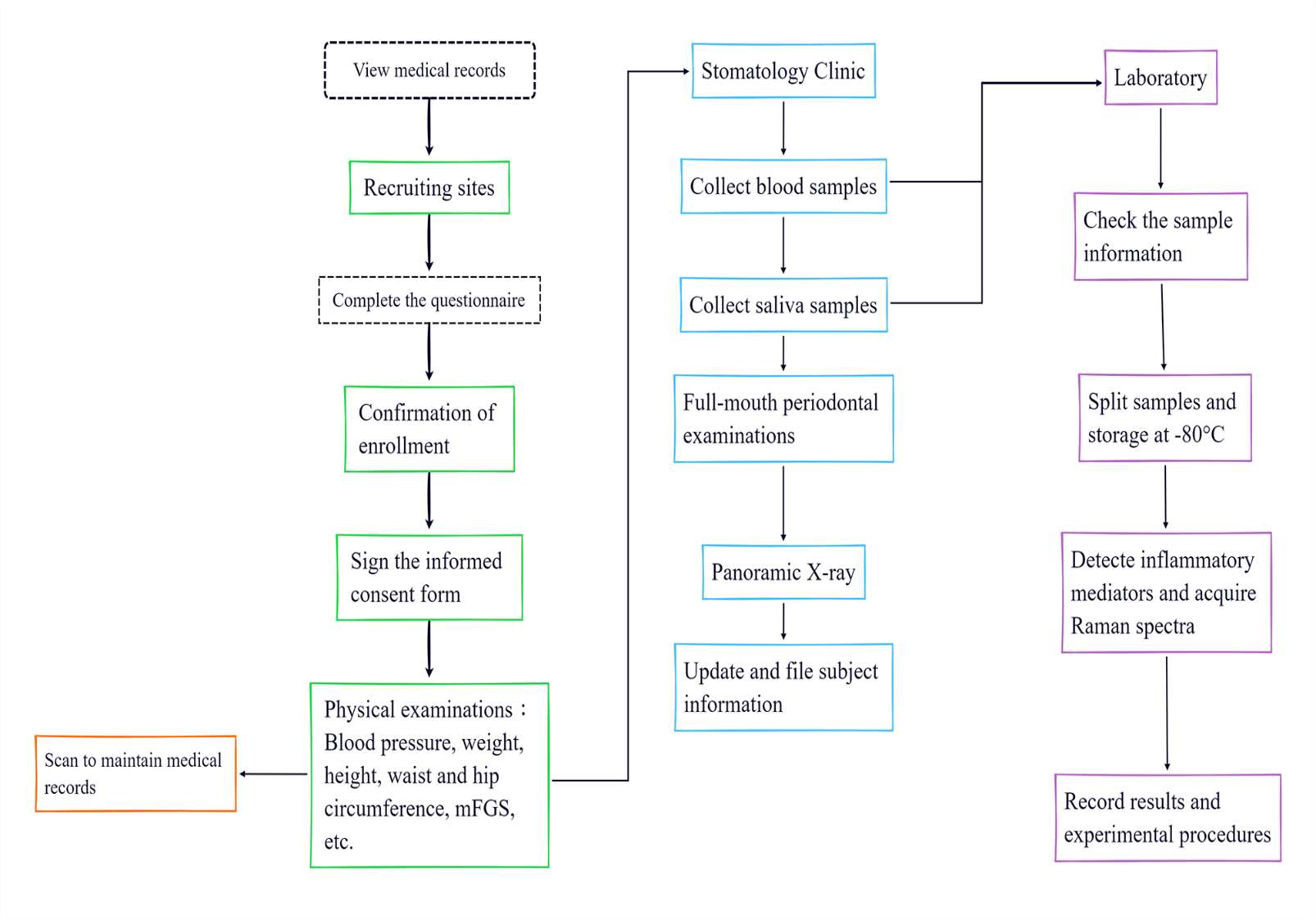


**Figure S1.** Flow chart of the whole procedure of subject recruitment, examination and sample measurement
