# Supplementary material for: Comparative analysis of serum and saliva samples using Raman spectroscopy: a high-throughput investigation in patients with polycystic ovary syndrome and periodontitis
Source: BMC Womens Health. 2023 Oct 4;23:522. doi: 10.1186/s12905-023-02663-y (PMC10552415; doi:10.1186/s12905-023-02663-y)
Supplement: Supplementary file 4 — Additional file 4: Table S1. The relevant script codes for R analysis. [file 12905_2023_2663_MOESM4_ESM.docx]

**Table S1.** The relevant script codes for R analysis

| **process** | **script code** |
| --- | --- |
| PCA | MyResult.pca1 = pca(serum, ncomp = 10, center = TRUE, scale = TRUE) # run pca method on data |
|  | par(mar=c(2,2,2,2)) |
|  | plot(MyResult.pca1) # barplot of the eigenvalues (explained variance per component) |
|  |  |
|  | MyResult.pca1 <- pca(serum, ncomp = 2,center = TRUE, scale = TRUE) |
|  | #plotIndiv(MyResult.pca2) |
|  | #plotVar(MyResult.pca2, cutoff = 0.8) |
|  | plotIndiv(MyResult.pca1, comp = c(1,2), |
|  | group = serum_group$PP1,ellipse = TRUE, |
|  | pch = as.factor(serum_group$PP1), |
|  | legend = TRUE, title = 'Score plot of PCA on serum Raman spectra', |
|  | legend.title = 'PP',X.label = 'comp1：36%', Y.label = 'comp 2：28%') |
| PLS-DA | x<- data.matrix(serum) |
|  | x1<- x[,-c(2:3)] |
|  | MyResult.plsda <- plsda(x1,serum_group$PP1,ncomp = 10) # 1 Run the method |
|  |  |
|  | plotIndiv(MyResult.plsda, comp = 1:2, |
|  | ind.names = FALSE, legend=TRUE, |
|  | ellipse = TRUE, title = 'Score plot of PLS-DA on serum Raman spectra', |
|  | X.label = 'comp1：31%', Y.label = 'comp 2：28%') |
|  | plotIndiv(MyResult.plsda, comp = 2:3, |
|  | ind.names = FALSE, legend=TRUE, |
|  | ellipse = TRUE, title = 'Score plot of PLS-DA on serum spectra') |
|  |  |
|  | set.seed(30) # for reproducbility in this vignette, otherwise increase nrepeat |
|  | perf.splsda.serum <- perf(MyResult.plsda, validation = "Mfold", folds = 5, |
|  | progressBar = FALSE, nrepeat = 10) # we suggest nrepeat = 50 |
|  | plot(perf.splsda.serum, col = color.mixo(5:7), sd = TRUE, legend.position = "horizontal") |
|  | perf.splsda.serum$choice.ncomp |
|  | auc.plsda <- auroc(MyResult.plsda,roc.comp = 1) |
|  | # grid of possible keepX values that will be tested for each component |
|  | list.keepX <- c(1:10, seq(20, 300, 10)) |
|  |  |
|  | # undergo the tuning process to determine the optimal number of variables |
|  | tune.splsda.serum <- tune.splsda(x1, serum_group$PP1, ncomp = 4, # calculate for first 4 components |
|  | validation = 'Mfold', |
|  | folds = 5, nrepeat = 10, # use repeated cross-validation |
|  | dist = 'max.dist', # use max.dist measure |
|  | measure = "BER", # use balanced error rate of dist measure |
|  | test.keepX = list.keepX, |
|  | cpus = 2) # allow for paralleliation to decrease runtime |
|  | plot(tune.splsda.serum, col = color.jet(4)) # plot output of variable number tuning |
|  | tune.splsda.serum$choice.ncomp$ncomp # what is the optimal value of components according to tune.splsda() |
|  | plotLoadings(MyResult.plsda,contrib = "max",comp=1, method = 'mean', ylim=100, |
|  | size.name = 0.6,size.legend = 2) |
|  | tune.splsda.serum$choice.keepX # what are the optimal values of variables according to tune.splsda() |
|  | optimal.ncomp <- tune.splsda.serum$choice.ncomp$ncomp |
|  | optimal.keepX <- tune.splsda.serum$choice.keepX[1:optimal.ncomp] |
|  |  |
|  | # form final model with optimised values for component and variable count |
|  | final.splsda.serum <- splsda(x1, serum_group$PP1, |
|  | ncomp = optimal.ncomp, |
|  | keepX = optimal.keepX) |
|  |  |
|  | plotIndiv(final.splsda.serum, comp = c(1,2), # plot samples from final model |
|  | group=serum_group$PP1, ind.names = FALSE, # colour by class label |
|  | ellipse = TRUE, legend = TRUE, # include 95% confidence ellipse |
|  | title = ' sPLS-DA on serum Raman spectra',X.label = 'comp 1：2%', Y.label = 'comp 2：16%') |
|  |  |
|  | plotIndiv(final.splsda.serum, comp = c(1,3), # plot samples from final model |
|  | group = serum_group$PP1, ind.names = FALSE, # colour by class label |
|  | ellipse = TRUE, legend = TRUE, # include 95% confidence ellipse |
|  | title = ' sPLS-DA on serum Raman spectra',X.label = 'comp 1：2%', Y.label = 'comp 3：5%') |
| ROC | auc.final.splsda.serum=auroc(final.splsda.serum,roc.comp= optimal.ncomp) |
| Permutational multivariate analysis of variance | library(vegan) |
|  | saliva.dist<-vegdist(saliva,method="gower") |
|  | hclust.saliva<-hclust(saliva.dist,method = "average") |
|  | plot(hclust.saliva) |
|  |  |
|  | saliva.pcoa <- cmdscale(saliva.dist, k=3, eig=T) |
|  | saliva.pcoa.points <- as.data.frame(saliva.pcoa$points) |
|  | sum_eig <- sum(saliva.pcoa$eig) |
|  | eig_percent <- round(saliva.pcoa$eig/sum_eig*100,1) |
|  | colnames(saliva.pcoa.points) <- paste0("PCoA", 1:3) |
|  | saliva.pcoa.result <- cbind(saliva.pcoa.points, salivaEnv) |
|  | head(saliva.pcoa.result) |
|  |  |
|  | library(ggplot2) |
|  | ggplot(saliva.pcoa.result, aes(x=PCoA1, y=PCoA2, color=salivaEnv$PP)) + |
|  | labs(x=paste("PCoA 1 (", eig_percent[1], "%)", sep=""), |
|  | y=paste("PCoA 2 (", eig_percent[2], "%)", sep="")) + |
|  | geom_point(size=4 |
|  | ) + stat_ellipse(level=0.6) + |
|  | theme_classic() |
|  |  |
|  | saliva.div <- adonis2(saliva~salivaEnv$PP,data = salivaEnv,permutations = 999,method="gower" ) |
|  | saliva.adonis <- paste0("adonis R2: ",round(saliva.div$R2,2), "; P-value: ", saliva.div$`Pr(>F)`) |
|  | library(ggalt) |
|  | ggplot(saliva.pcoa.result, aes(x=PCoA1, y=PCoA2, color=salivaEnv$PP, group = salivaEnv$PP)) + |
|  | labs(x=paste("PCoA 1 (", eig_percent[1], "%)", sep=""), |
|  | y=paste("PCoA 2 (", eig_percent[2], "%)", sep=""), |
|  | title=saliva.adonis) + |
|  | geom_point(size=5) + |
|  | geom_encircle(aes(fill=salivaEnv$PP), alpha = 0.1, show.legend = F) + |
|  | theme_classic() + coord_fixed(1) |
|  |  |
|  | algo = c("manhattan", "euclidean", "canberra", "bray", "kulczynski", "jaccard", "gower", "altGower", "morisita", "horn", "mountford", "raup", "binomial", "chao", "cao", "mahalanobis") |
|  | p=c() |
|  | for(title in algo) |
|  | { |
|  | res = adonis(saliva ~ salivaEnv$PP, data = salivaEnv, |
|  | distance = title, permutations = 999) |
|  |  |
|  | p = c(p, res$aov.tab$Pr[1]) |
|  | print(title) |
|  | } |
|  | perm = data.frame(algo, p) |
|  |  |
|  | anosim.result<-anosim(saliva.dist,salivaEnv$PP,permutations = 999) |
|  | summary(anosim.result) |
|  | plot(anosim.result, col = c('#FFD700','#FF7F00','#EE2C2C')) |
| correlation heat map | spearHH <- T0_Spearmancorrelation_HH_data[,-1] |
|  | corspearHH <- round(cor(spearHH, method = 'spearman'), 3) |
|  | head(corspearHH[,1:6]) |
|  | library(ggcorrplot) |
|  | ggcorrplot(corspearHH) |
|  |  |
|  | ggcorrplot(corspearHH,method = "circle",lab=T) |
|  |  |
|  | pspearHH <- cor_pmat(spearHH) |
|  | ggcorrplot(corspearHH,hc.order = T, |
|  | ggtheme = ggplot2::theme_void(base_size = 15), |
|  | colors = c("CornflowerBlue","white","Salmon"), |
|  | lab = T,lab_size = 2, |
|  | tl.cex = 8, |
|  | p.mat = pspearHH, |
|  | sig.level = 0.05, |
|  | pch = 4, |
|  | pch.cex = 10) |
|  |  |
|  | ggcorrplot(corspearHH, method = "circle", type = "full", ggtheme = ggplot2::theme_void, |
|  | show.legend = TRUE, legend.title = "Corr", show.diag = T, |
|  | colors = c("#839EDB", "white", "#FF8D8D"), outline.color = "white", |
|  | hc.order = T, hc.method = "complete", lab = TRUE, lab_col = "black", |
|  | lab_size = 4, p.mat = pspearHH, sig.level = 0.05, insig = "blank", pch = 4, pch.col = "black", pch.cex = 3, tl.cex = 8, |
|  | tl.col = "black", tl.srt = 45, digits = 2) |
