# Supplementary material for: Comparative analysis of serum and saliva samples using Raman spectroscopy: a high-throughput investigation in patients with polycystic ovary syndrome and periodontitis
Source: BMC Womens Health. 2023 Oct 4;23:522. doi: 10.1186/s12905-023-02663-y (PMC10552415; doi:10.1186/s12905-023-02663-y)
Supplement: Supplementary file 5 — Additional file 5: Table S2. Demographic data, anthropometric and periodontal indicators in all 88 subjects. [file 12905_2023_2663_MOESM5_ESM.docx]

**Table S2.** Demographic data, anthropometric and periodontal indicators in all 88 subjects

| **Parameters** | **Results (n=88)** |
| --- | --- |
| Demographic characteristics |  |
| Age (years) | 25.9±3.3 |
| Education |  |
| lower than university level | 22 (25) |
| university level or higher | 66 (75) |
| Income (CNY) |  |
| <9,000 | 38 (43.2) |
| ≥9,000 | 50 (56.8) |
| Smoking |  |
| Never | 86 (97.7) |
| Previously | 2 (2.3) |
| Drinking |  |
| Never | 78 (88.6) |
| Seldom | 10 (11.4) |
| Regular Dental Visit |  |
| No | 61 (69.3) |
| Yes | 27 (30.7) |
| BOB |  |
| No | 52 (59.1) |
| Yes | 36 (40.9) |
| Anthropometric indicators |  |
| BMI (kg/m^2^) | 22.40±3.71 |
| WHR | 0.77±0.05 |
| SBP (mmHg) | 116.4±11.7 |
| DBP (mmHg) | 71.5±9.0 |
| HR (bpm) | 84.7±12.9 |
| mFGS | 1.8±1.5 |
| Periodontal status |  |
| Periodontal health | 28 (31.8) |
| Gingivitis | 32 (36.4) |
| Stage I | 14 (15.9) |
| Stage II | 14 (15.9) |
| Periodontal parameters |  |
| PD (mm) | 1.63 (1.51, 2.00) |
| FMPS (%) | 71.0 (56.0, 86.8) |
| ≤50 | 17 (19.3) |
| >50 | 71 (80.7) |
| BOP (%) | 12.58 (7.74, 31.51) |
| <10 | 28 (31.8) |
| 10-50 | 55 (62.5) |
| >50 | 5 (5.7) |
| PD≥4mm (%) | 0.0 (0.0, 2.8) |
| CAL1-2mm (%) | 0.0 (0.0, 2.8) |

Data were presented as mean ± SD or median (IQR) or frequency (%).
