# Supplementary material for: Comparative analysis of serum and saliva samples using Raman spectroscopy: a high-throughput investigation in patients with polycystic ovary syndrome and periodontitis
Source: BMC Womens Health. 2023 Oct 4;23:522. doi: 10.1186/s12905-023-02663-y (PMC10552415; doi:10.1186/s12905-023-02663-y)
Supplement: Supplementary file 6 — Additional file 6: Table S3. Comparison of demographic data, anthropometric, hormonal and inflammatory indicators between the periodontitis and non-periodontitis groups. [file 12905_2023_2663_MOESM6_ESM.docx]

**Table S3.** Comparison of demographic data, anthropometric, hormonal and inflammatory indicators between the periodontitis and non-periodontitis groups

| **Parameters** | **Periodontitis (n=28)** | | **Non-Periodontitis (n=60)** | ***P* value** |
| --- | --- | --- | --- | --- |
| Age (years) | | 26.2±3.2 | 25.8±3.4 | 0.543 |
| Education | |  |  |  |
| lower than university level | | 10(35.7) | 12(20) | 0.113 |
| university level or higher | | 18(64.3) | 48(80) |  |
| Income (CNY) | |  |  | 0.674 |
| <9,000 | | 13(46.4) | 24(40) |  |
| ≥9,000 | | 15(53.6) | 35(60) |  |
| Smoking | |  |  | 0.462 |
| Never | | 28 (100.0) | 58 (96.7) |  |
| Previously | | 0 (0.0) | 2 (3.3) |  |
| Drinking | |  |  | 0.323 |
| Never | | 26 (92.9) | 52 (86.7) |  |
| Seldom | | 2 (7.1) | 8 (13.3) |  |
| Regular Dental Visit | |  |  | 0.430 |
| No | | 21 (75.0) | 40 (66.7) |  |
| Yes | | 7 (25.0) | 20 (33.3) |  |
| Dietary habits | |  |  | 0.456 |
| Fat-reduced or normal meals | | 20(71.4) | 38(63.3) |  |
| high-fat meal | | 8(28.6) | 22(36.7) |  |
| BOB | |  |  | < 0.001 |
| No | | 6 (21.4) | 46 (76.7) |  |
| Yes | | 22 (78.6) | 14 (23.3) |  |
| BMI (kg/m^2^) | | 21.71 (20.57, 25.4166) | 21.16 (19.16, 24.31) | 0.131 |
| WHR | | 0.79±0.05 | 0.77±0.05 | 0.045 |
| SBP (mmHg) | | 117.0±12.0 | 116.1±11.7 | 0.725 |
| DBP (mmHg) | | 71.0±7.6 | 71.7±9.6 | 0.729 |
| HR (bpm) | | 88.5±14.2 | 83.0±12.0 | 0.061 |
| mFGS | | 2.0 (0.0,3.0) | 1.0 (0.0,3.0) | 0.993 |
| PD (mm) | | 2.15(2.02,2.50) | 1.56(1.47,1.64) | < 0.001 |
| Percentage of sites with PD ≥4 mm (%) | | 7.0(3.0,13.8) | 0(0,0) | < 0.001 |
| Percentage of sites with PD ≥6 mm (%) | | 0(0,0) | 0(0,0) | 0.143 |
| FMPS (%) | | 91(77,100) | 61(50,77) | < 0.001 |
| BOP (%) | | 37.2(31.5,47.2) | 10.1(7.1,13.4) | < 0.001 |
| TG (mmol/L) | | 0.70(0.57,1.12) | 0.86(0.60,1.40) | 0.033 |
| TC (mmol/L) | | 4.87±0.89 | 4.62±0.78 | 0.249 |
| LDL (mmol/L) | | 2.85(2.50,3.54) | 2.63(2.40,3.11) | 0.603 |
| HDL (mmol/L) | | 1.57(1.04.1.74) | 1.38(1.17,1.65) | 0.488 |
| FPG (mmol/L) | | 4.90(4.77,5.30) | 5.01(4.70,5.22) | 0.977 |
| FSH (IU/L) | | 6.77 (5.87, 7.53) | 7.34 (6.03, 8.62) | 0.094 |
| LH (IU/L) | | 9.80 (5.39, 14.38) | 11.68 (6.20, 19.71) | 0.111 |
| LH/FSH | | 1.74 (0.77, 2.05) | 2.01 (0.86, 2.69) | 0.713 |
| T (μg/L) | | 0.73±0.29 | 0.70±0.23 | 0.648 |
| PRL (μg/L) | | 14.41 (11.60,17.50) | 14.39 (8.73, 24.27) | 0.674 |
| P (nmol/L) | | 0.74 (0.42,1.10) | 0.81 (0.40,1.13) | 0.872 |
| E2 (pmol/L) | | 37.79 (30.65,53.36) | 40.30 (30.17,63.25) | 0.844 |
| se IL-6 (pg/mL) | | 2.82±2.13 | 2.56±1.67 | 0.523 |
| se IL-17A (pg/mL) | | 20.23(11.81,235.61) | 26.40(12.08,1319.10) | 0.385 |
| se MMP-8 (ng/mL) | | 5.08(2.64,8.94) | 4.09(2.19,8.67) | 0.507 |
| sa IL-6 (pg/mL) | | 1.94(1.02,4.49) | 2.54(1.66,4.43) | 0.358 |
| sa IL-17A (pg/mL) | | 10.39(5.95,13.91) | 9.82(7.25,15.94) | 0.774 |
| sa MMP-8 (ng/mL) | | 345.36(189.54,535.88) | 73.64(33.49,175.75) | < 0.001 |

Data are presented as mean ± SD or median (IQR) or frequency (%).
