# Supplementary material for: Comparative analysis of serum and saliva samples using Raman spectroscopy: a high-throughput investigation in patients with polycystic ovary syndrome and periodontitis
Source: BMC Womens Health. 2023 Oct 4;23:522. doi: 10.1186/s12905-023-02663-y (PMC10552415; doi:10.1186/s12905-023-02663-y)
Supplement: Supplementary file 7 — Additional file 7: Table S4. Attribution of the major peaks obtained from Raman analysis of serum and saliva samples (± 8 cm−1). [file 12905_2023_2663_MOESM7_ESM.docx]

**Table S4.** Attribution of the major peaks obtained from Raman analysis of serum and saliva samples (± 8 cm^−1^)

| **Peaks(cm^-1^)** | **Target compounds** | **Vibrational mode** | **References** |
| --- | --- | --- | --- |
| 747 | thymine | ring breathing mode of DNA/RNA bases | [44] |
| 752 | hemoglobin |  | [44] |
| 996 | phenylalanine | aromatic ring breathing, C–CH_3_ rocking motions | [39] |
| 1118 | lipid | C-C stretch | [43] |
| 1146 | lipid | C-C skeletal stretching | [43] |
| 1255 | proteins | C-H bending mode, amide III | [43] |
| 1300 | lipid, fatty acid | CH_2_ twisting | [44] |
| 1324 | proteins | Amide III | [42] |
| 1328 | guanine, adenine | C–H deformation | [41] |
| 1332 | - | -C stretch of phenylalanine | [44] |
| 1437 | - | C–H deformation | [44] |
| 1439 | collagen | CH_3_, CH_2_ deformation vibrations | [44] |
| 1507 | β-carotene | C=C stretching mode | [44] |
| 1573 | protein |  | [44] |
| 1646 | protein | amide I band | [44] |
| 1652 | protein | CO stretching mode, amide I | [40] |
| 2916 | lipids | C-H (CH_2_) stretching/C-H (CH_3_) stretching | [44] |
| 2918 | lipid | CH_3_ stretching vibrations | [44] |
| 2920 | protein | CH_3_ stretching vibrations | [44] |
